# Mechanics of Pure Bending and Eccentric Buckling in High-Strain Composite Structures

**DOI:** 10.3390/ma17040796

**Published:** 2024-02-07

**Authors:** Jimesh D. Bhagatji, Oleksandr G. Kravchenko, Sharanabasaweshwara Asundi

**Affiliations:** Department of Mechanical and Aerospace Engineering, Old Dominion University, Norfolk, VA 23529, USA; sasundi@odu.edu

**Keywords:** composite boom, bending characterization, buckling, continuum damage mechanics

## Abstract

To maximize the capabilities of nano- and micro-class satellites, which are limited by their size, weight, and power, advancements in deployable mechanisms with a high deployable surface area to packaging volume ratio are necessary. Without progress in understanding the mechanics of high-strain materials and structures, the development of compact deployable mechanisms for this class of satellites would be difficult. This paper presents fabrication, experimental testing, and progressive failure modeling to study the deformation of an ultra-thin composite beam. The research study examines the deformation modes of a post-deployed boom under repetitive pure bending loads using a four-point bending setup and bending collapse failure under eccentric buckling. The material and fabrication challenges for ultra-thin, high-stiffness (UTHS) composite boom are discussed in detail. The continuum damage mechanics (CDM) model for the beam is calibrated using experimental coupon testing and was used for a finite element explicit analysis of the boom. It is shown that UTHS can sustain a bending radius of 14 mm without significant fiber and matrix damage. The finite element model accurately predicts the localized transverse fiber damage under eccentric buckling and buckling stiffness of 15.6 N/mm. The results of the bending simulation were found to closely match the experimental results, indicating that the simulation accurately shows deformation stages and predicts damage to the material. The findings of this research provide a better understanding of the structure characteristics with the progressive damage model of the UTHS boom, which can be used for designing a complex deployable payload for nano-micro-class satellites.

## 1. Introduction

Composites were used as a building block of the traditional satellite that debuted in the early 1970s. It was used on the Apollo capsule as an Avcoat ablative heat shield, fiberglass honeycomb structure [1], etc. Since then, advanced composites have been the choice of various space programs such as reusable launch vehicles, observation satellites, and the International Space Station (ISS) [2]. Fast-forward to today where advanced composites still play a significant role in the advancement of space programs. Emerging small satellite technology also cannot escape the use of composites. Composite materials are used in various avenues of small satellite technology like solar structure panels [3], high gain antenna [3], momentum/reaction wheel [4], deorbiting [5], etc. These composite structures are typically used in rigid structural elements, while novel nano- and micro-class satellites, which are constrained by size, weight, and power, require a new class of high-strain composite structures. The small satellite technology demands a high post-deployment surface area to packaging volume as well as compact deployable mechanisms, which require further advancement in the high-strain composite structures.

The early development of the boom started with simpler deployable structures with a tape spring [6] cross-section made of Carbon Fiber-Reinforced Polymer (CFRP). Composites enabled adaptive performance, a coefficient of thermal expansion, and reduced mass. Furthermore, various cross-section booms were attempted in order to improve the structure performance of the boom, considering increasing the (stored) packaging volume. Tubular Extendible Member (STEM) booms [7] and tape springs [6] are characterized under a single-shell storable boom and Triangular Rollable and Collapsible (TRAC) booms [8], lenticular shearless booms, and Collapsible Tubular Mast (CTM) boom [9] are characterized as double-shell storable booms. The selection of the boom is highly subjective to the loading conditions for the payload. A parametric analysis [10] was conducted to identify the optimal boom geometries that maximize stiffness for various cross-sections of boom. Results indicated that the CTM provides a structural advantage with a maximum second moment of inertia in all three axes when compared among the various double-shell booms.

NASA has been investigating the performance of CTM for the Advanced Composite Solar Sail System (ACS3) [11] technology demonstration mission. To develop high-strain composite structures with improved packaging efficiency and deployed structural performance, accurate prediction of strain-stress states and failure modes of flexural members composed of thin composite laminates is necessary. To explore this potential benefit, various test setups [12,13,14] have been conducted on flat coupons of woven-ply CFRP materials subjected to pure moments. In typical flat coupon bending, the high-strain composite material exhibits fiber tensile stiffening and compression softening, with a net effect that leads to a gradual decrease in bending stiffness as strain increases. The advantage of using this method was its simplified experimental setup; however, under high deformation structure bending of UTHS/CTM composite, the material experiences a bi-directional strain that was not accurately captured by the current test setup.

In contrast to flat coupon bending, an experimentally intensive technique [15] can be used to formulate a stress-based failure criterion in terms of failure parameters. This approach considers a repeating unit cell of a symmetric two-ply plain weave laminate and the stress resultants from a homogenized plate model. Five sets of tests were conducted to estimate the failure parameters, and five additional combined loading test configurations were tested for validation. This approach has the advantage of finding a failure locus for a two-ply plain weave laminate in terms of force and moment resultants, making a six-dimensional loading space with an experimental intensive approach.

It is essential to find the bending characteristics of the deployable composite boom to create an effective design. A simple and precise approach needs to be taken to bridge this gap. Few studies, such as [16], observed contradictory modeling results from the experimental results due to an inappropriate methodology to determine the flexural modulus of the material for boom flattening lengths in the range of 40–100 mm. For higher flattening lengths of 250–500 mm with a material thickness range of 0.2–0.4, the material remains in the elastic region under large deformation structure bending. It was important to understand the relation between the size factor of UTHS/CTM composite boom and stress in the material. Another study [17] conducted a flattening test for the deployable composite boom of a flattening length (approx.) 280 mm, where nonlinearity was observed mostly due to geometry rather than to material nonlinearity itself, which can include plasticity and localized damage. Gaining a precise knowledge of strain behavior and non-linear effects under large deformation loads will allow for the design of the deployable structures more effectively, preventing potential failure.

This paper presents analysis, design, and fabrication approach of UTHS/CTM structure for small satellite applications and validates its performance. The boom structure is a basic building block for in-space assembly and Lunar exploration missions. They offer structural support and stability for post-deployment payload assembly in low packaging volume for in-space assembly of telescope or antenna [18]. Similarly, in lunar exploration, booms are used as deployable towers for critical instruments/equipment for power, communication, and weather research [19]. It is important to understand structural failure behavior post-deployment and determine safety factors for such deployable payloads. Hence, it is essential to develop predictive modeling tools that can consider the mechanical behavior under bending and buckling of thin booms in the post-deployed configuration.

The first section discusses the composite material and design of the lenticular cross-section boom (CTM), as well as elaborates on the fabrication process and challenges that were addressed to develop a consistent deployable structure. The structural level testing of the boom was investigated for the large deformation behavior of the composite boom using four-point bending tests and coupled bending-buckling behavior through an eccentric compression test. The large deformation behavior was used to evaluate damage progression during pure bending and to determine the critical bending radius. This analysis was crucial for understanding the folding deformation and the associated damage that occurs during rolling. Moreover, complex deployable mechanisms [3] often subject UTHS booms to intricate loading conditions, leading to localized bending failure. Therefore, it becomes crucial to comprehend the deformation behavior under short spans to simulate localized pure bending.

Additionally, another mechanical test was conducted where the coupled bending and compression behavior was assessed using an eccentric buckling setup. This test allowed for determining global structural failure during post-deployment loading conditions, where the deployed UTHS boom underwent compressive loading with pure bending deformation. In the second section, an experimental approach for calibrating finite element analysis (FEA) Continuum Damage Mechanics (CDM) model is discussed, which used 0° and 45° coupon tensile testing of the weave. The validation of FEA CDM of the boom was performed using both four-point bending and eccentric buckling by comparing with the experimental results. Modeling results of four-point bending tests and eccentric buckling of ultra-thin composites agreed closely with the mechanical test data. FE analysis revealed that during the snapping of the boom, no significant damage was induced in the material, allowing for a reliable UTHS boom deployment. In the case of eccentric buckling, FEA accurately captured the global buckling with transverse fiber damage. The proposed material and structural testing, along with modeling methodology, can be adopted to the UTHS boom for the various deployable payloads used in small-class satellites and in space structures.

## 2. Material and Fabrication for Mechanical Test

### 2.1. Materials

To create high-deformation structures, it was necessary to select a material that was both lightweight and able to withstand large non-linear deformation of UTHS boom. Carbon fiber was chosen as the material in this case due to its excellent strength-to-weight ratio. However, in contrast to glass fiber, carbon fiber provides better strength but at a lower strain, which opposes the high-strain material requirement.

Therefore, thin laminates of only one or two plies were of particular interest in our study to meet the material requirement. To maximize the laminate surface strain and axial modulus (E_1_) of the laminate, a single-ply twill-weave was selected. Twill weave at a 45° weave orientation provides high shear strain and better formability during manufacturing, especially on curved surfaces. To simplify the handling of the material during manufacturing, high-quality carbon fiber prepreg 3 K (200 gsm), 2 × 2 twill weave was procured from Fibreglast, Brookville, OH, USA.

Coupon testing was carried out for both single-ply weave and two-ply weave before deciding to use the single-ply weave, as presented later in the paper. Uniaxial tension tests of a 0° weave laminate and 45° weave laminate were prepared from weave laminate panels. These tensile specimens were cut into dogbone specimens of 20 mm width and 200 mm long (including tabs) using a ProtoMAX waterjet system by Omax, Kent, WA, USA. These specimens were tested in an MTS Alliance RF/300 machine with a load cell of 300 kN capacity by MTS System, Eden Prairie, MN, USA. Testing was conducted with a crosshead displacement of 1 mm/min. The test was recorded using a GOM 3D Digital Image Correlation (DIC) system to accurately capture the strain distribution during testing.

### 2.2. Composite Boom Fabrication

**Figure 1:**
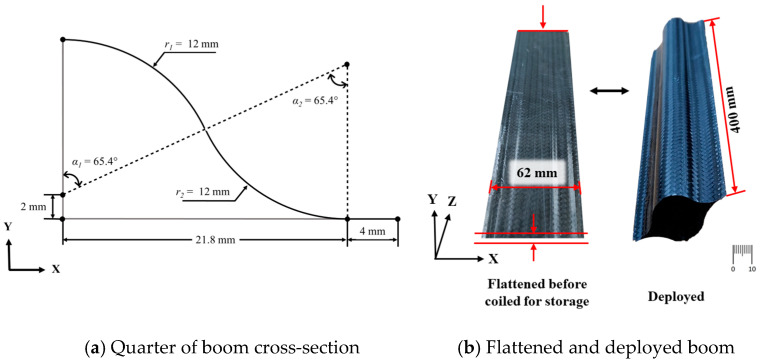
Design parameters of the UTHS/CTM deployable structure.

The cross-section of the specimen can be parametrized into six independent parameters, as shown in Figure 1a. Here, in order to reduce the inconsistency of in-plane strain along the cross-section during flattening and wrapping:

1. *α*_1_ and *α*_2_ were selected as same value (*α*_1_ = *α*_2_ = *α*).
2. *r*_1_ and *r*_2_ were selected as same value (*r*_1_ = *r*_2_ = *r*).

The design of the specimen has been simplified to three parameters—*α*, *r*, and *t*. These parameters determine the structural properties of the composite boom. The selection of these three parameters was primarily determined by the bending radius of the composite structure (boom), flattening height, and second moment of area, which were obtained from the mission requirements. The design of the optimal boom must consider the stability of its structure under post-deployment loading conditions and its ability to fit within the volume constraints specified by the mission requirements. In this study, Old Dominion University’s 3U CubeSat constraints were considered [20]. The final selected design parameters are shown in Figure 1a. With these parameters, the structure has a second moment of inertia of 2.78 × 10^4^ mm^4^, with a flattened height of 62 mm, shown in Figure 1b.

**Figure 2:**
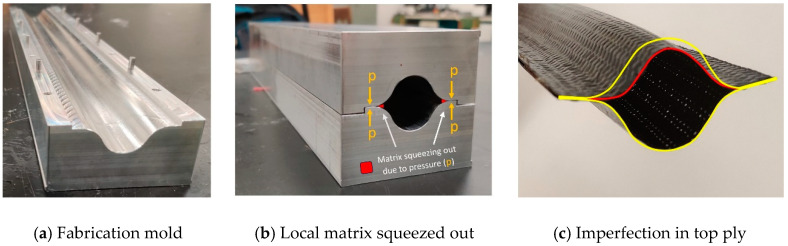
Fabrication of composite boom.

This lenticular cross-section was achieved by joining a flat end (web) of two halves (omega-shaped) made of carbon fiber prepreg twill weave. In the literature [9,11], a rigid aluminum or rubber internal mold was employed for the fabrication of the boom, alongside two split molds. However, this approach requires additional tooling to support the internal surface of the boom. Hence, we decided to examine the co-cured boom structure using internal vacuum bagging with two split molds. Multipurpose 6061 Aluminum order from McMaster-Carr, Elmhurst, Illinois, USA was used to fabricate 400 mm long mold as shown in Figure 2a. Low tolerance was provided in the mold assembly for the alignment of two halves when closed. One of the key requirements for the deployable structures is the ability to withstand the high bending strain of the boom structure, which does not produce large non-linear deformation in the composite material, similar to Von Karman non-linear beam model [21] and Carrera Unified Formulation (CUF) [22]. A single layer was selected for this study, as it can provide the necessary bending strain and stiffness demanded by the structure. A 45° angle woven orientation was selected to improve the structure’s bending flexibility, as well as to provide additional benefits in terms of torsional stiffness and in-plane shear stiffness.

A single cure cycle is recommended by the manufacturer at 155 °C (310 °F) at a ramp at 5 °C/min with a hold stage for 120 min and subsequent cool down. During the early iterations of the process, a simplified manufacturing process was first conducted without vacuum bagging. The layup prepreg sheets were laid down on the two-half mold and compressed against the mold using the hand roller to obtain the desired lenticular shape. Then, the mold was carefully closed and placed in an autoclave for a single-stage cure cycle at 80 Psi pressure. However, this process resulted in a significant deviation from the desired geometry due to the absence of internal support for the top woven ply. As the temperature increased, the viscosity of the epoxy resin reduced, resulting in the peeling of the unsupported ply from the mold surface. This caused the deformed lenticular shape as shown in Figure 2c where the upper ply was in a deformed shape (in red) in comparison to the bottom ply. This issue was resolved by providing internal support to the top ply during the curing process by using internal support provided by the vacuum bag film, which was used as a sleeve and was inserted into the hollow space of the lenticular shape. This process provided the desired lenticular shape but was not able to achieve uniform thickness near the bond line of two halves of the boom. Upon close observation of the sample, an accumulation of epoxy resin was found near the web as shown in Figure 2b. As the two ends of the lenticular shape were joined with the help of mold pressure, the matrix tended to squeeze out [23,24] at elevated temperatures. This accumulated epoxy resin near the web resulted in a local increase in thickness, which upon bending resulted in cracks. To solve the issue of non-uniform thickness, a co-curing cycle was adopted. The initial cure cycle allowed for the matrix to partially cure by reaching the gelation point. Therefore, during the co-cure stage, when the mold was closed, matrix squeezing was prevented. As a result, a uniform thickness joint was achieved between the two halves of the boom. In summary, the proposed two-stage co-curing process with an inner vacuum bag offered an alternative fabrication method with simplified tooling by eliminating the need for an internal mold. This approach provided uniform pressure on the inner surface of the lenticular shape and prevented matrix squeezing near the web, which was observed when two-piece mold was used (Figure 2b).

### 2.3. Mechanical Testing

The damage in the material is mainly by bi-directional strains during localized boom bending. It was crucial to quantify the damage to the composite boom caused by large deformation bending, which can cause the matrix and the fiber damage due to the complex local state of stress. To evaluate the large deformation capability of the boom structure, four-point bending testing was conducted on the boom with a 160 mm span. A load–displacement plot was chosen for analyzing bending performance to avoid the need for calculating varying moments of inertia or centroid shift. This approach also simplifies the comparative analysis of experimental data with reaction force versus displacement plots in FEA simulations.

To understand the potential of local damage in the material during the rolling of the boom, two loading cycles were performed during four-point bending. Upon bending for two successive cycles, any damage that was caused by the first bending will be revealed in the load vs. deformation plot as reduced bending stiffness and potential reduction in the critical load.

**Figure 3:**
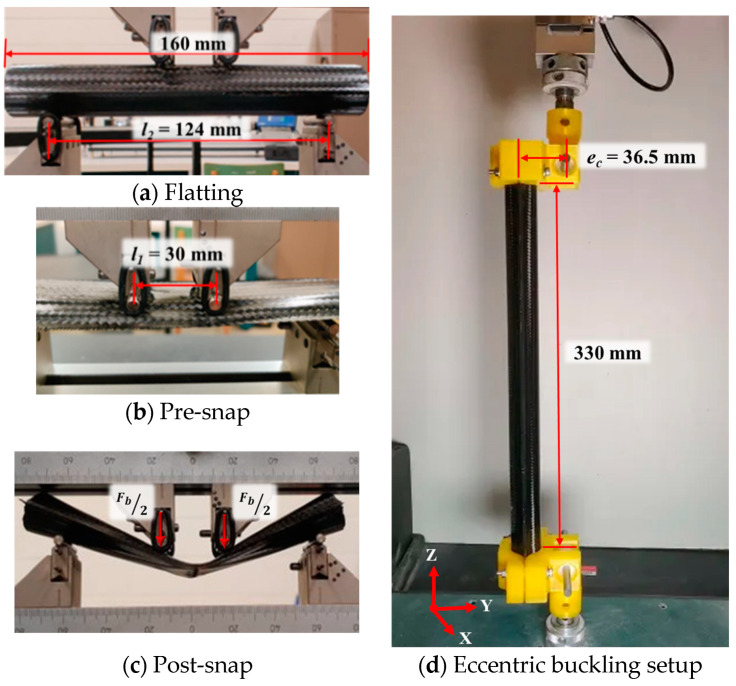
(**a**–**c**) Four-point bending test setup and deformation modes. (**d**) Custom 3D printed end attachment for eccentric buckling.

The fixed bottom roller span was 124 mm of four-point bending setup (as shown in Figure 3a). To experimentally test the effect of varying critical radius of curvature upon local bending, the UTHS boom specimens were evaluated under with varying top roller span lengths: 20, 30, and 50 mm using a 10 kN MTS test machine by MTS System, Eden Prairie, MN, USA. (as shown in Figure 3b). The different spans were considered to understand any damage with varying bending curvature would in the boom. A preload was applied to the boom to avoid boom to roll over and capture the large deformation of the boom without slipping and provided a constant moment at the center of the boom. To demonstrate localized high-strain deformation of the boom, a crosshead was displaced to induce a strain of 2% in the structure when subjected to bending.

The UTHS boom was also tested under coupled buckling (*F_c_*) and bending (*M_c_ = F_c_* × *e_c_*) conditions to simulate post-deployment global bending scenarios through an eccentric buckling test. A custom 3D printed end attachment was designed to feature fixed end condition at the UTHS boom and bearing-supported load-cell adapter, eccentrically positioned at *e_c_* = 36.5 mm from the center of the boom. A displacement-controlled eccentric load was applied until failure at a rate of 1 mm/min on a 330 mm long boom.

## 3. Modeling of Ultra-Thin Composite Beam

To develop and test a design strategy for ultra-thin composite booms, CDM material model was validated and used for non-linear analysis of the UTHS boom bending simulation. The FEA modeling was validated using experimental results on the coupon scale and using composite boom geometry. The CDM material model of composite boom allowed to capture the non-linear material behavior during large deformation experienced in four-point bending.

### 3.1. Material Model Calibration

A progressive damage analysis (PDA) was performed using a finite element model discussed in the following section to determine potential failure modes in composite material during the boom bending. The CDM model discussed in [25] was used for fabric-reinforced composites with a non-linear response to matrix shear, assuming orthogonal fiber directions and using orthotropic damaged elasticity for in-plane stress-strain relations shown in Equation (1). This analysis used CDM to model damage in warp and weft directions, as well as matrix damage. Within this framework, the damage variables *d*_1_ and *d*_2_ were utilized to represent fiber damage in the warp and weft directions, respectively. These variables effectively capture the extent of damage resulting from both tensile and compressive loading. The analysis also incorporated matrix shear plasticity by introducing the matrix damage variable *d*_12_. This variable accounts for the extent of damage in the matrix material due to shear forces.

(1) $$\left[\;\begin{array}{ccc} \frac{1}{\left(1-d_{1}\right)E_{1}} & \frac{{-v}_{12}}{E_{1}} & 0\\ \frac{{-v}_{21}}{E_{2}} & \frac{1}{\left(1-d_{2}\right)E_{2}} & 0\\ 0 & 0 & \frac{1}{\left(1-d_{12}\right)2G_{12}} \end{array}\;\;\right]\;\;\left[\;\begin{array}{c} \sigma_{11}\\ \sigma_{22}\\ \sigma_{12} \end{array}\;\right]=\left[\;\begin{array}{c} \varepsilon_{11}\\ \varepsilon_{22}\\ \varepsilon_{12}^{el} \end{array}\;\right]$$

**Figure 4:**
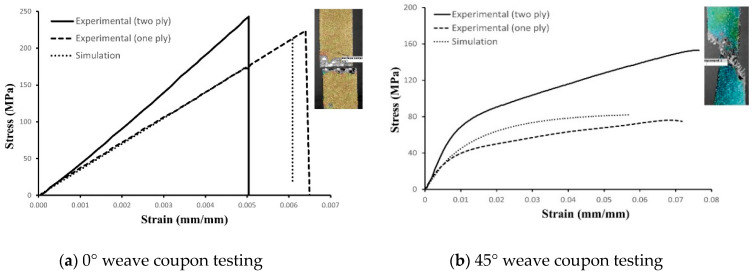
Experimental testing results of uniaxial tension test.

The CDM material model was accessed by creating material with suffix ABQ_PLY_FABRIC, an embedded user subroutine (VUMAT) in Abaqus/Explicit 2021 was used to model woven ply. The stress component of FEM elements is transferred onto the fiber failure criteria, which updates the damage threshold to determine the fiber damage activation function. The damage threshold satisfies the Kuhn–Tucker complementary conditions, ensuring its monotonically increasing behavior [25]. By utilizing the damage threshold and fracture energy, the fiber damage variable is determined. Figure 4 presents the test results obtained from the uniaxial tension test of coupons used to calibrate the CDM model. A difference in modulus and strength was observed between single-ply and double-ply laminates for both 0° weave laminate and 45° weave laminate.

The double-ply laminate shows minimal to no pinholes (voids), leading to increased modulus and strength. On the contrary, the single-ply laminate had detected pinholes, affecting its properties. A micromechanical model [26] with pinholes demonstrates a similar increase in effective mechanical properties as the number of ply increases. However, for this study, a single layer was chosen based on high-deformation structure requirements, hence, despite the presence of pinholes, single-ply laminate’s equivalent properties were used to calibrate the material model.

The elastic modulus (*E*_1_) and strength (*X*_1_) of the CDM material model were calibrated using a uniaxial tension test of a 0° weave laminate, resulting in values of 36.9 GPa and 240 MPa, respectively, as shown in Figure 4a. These effective properties are relatively low compared to the properties of carbon fiber warp/weft tows, and can be attributed to the twill weave architecture. Notably, the effective mechanical properties obtained from the micromechanical model [26] align well with the properties determined through tensile experiment.

Shear modulus (*G*_12_) was calculated using Equation (2), resulting in values of 1.56 GPa, where *E_x_* is elastic modulus and *v_xy_* is Poisson’s ratio, determined from the stress-strain curve of 45° weave coupon (shown in Figure 4b). Shear damage threshold (*S*) and initial effective shear yield stress ($\sigma_{y}^{0}$) are calculated using Equation (3), resulting in values of 15 MPa and 25 MPa, where *σ_e_* is the elastic limit and *σ_y_* is the yield strength of the 45° weave coupon.

(2) $$G_{12}=\frac{Ex}{2(1+v_{xy})}$$

(3) $$\;\;\;S=\frac{\sigma_{e}}{2},\;\sigma_{y}^{0}=\frac{\sigma_{y}}{2}$$

The shear hardening behavior of the material is characterized by two parameters: the coefficient of plastic hardening (*C*) and the power term of plastic hardening (*p*). These parameters were calibrated using uniaxial tension tests on 45° weave coupons to curve fit data with the shear hardening function [25]. The fracture toughness properties needed for the CDM material model in FEA were established based on Refs. [27,28], and are presented in Table 1.

To validate the calibrated material model, a virtual coupon was simulated using FEA. Figure 4 also presents the simulation results for the 0° and 45° weave coupons, demonstrating fiber and shear failure, respectively, in agreement with the results of the tensile test. Figure 4a shows that the simulation data for the 0° weave coupon match the experimental data, while the 45° weave coupon exhibits a slight offset in the shear plastic zone despite a good match in initial modulus. Given that the applied strain for bending is limited to 2%, this calibration remains satisfactory for further analysis.

### 3.2. Boom Finite Element Simulation

This section presents a bending simulation of a composite boom under high deformation, using both linear elastic model and the CDM material model. The linear elastic analysis was used to determine the (reaction) force–displacement plot in the composite boom, while the PDA was used to evaluate the damage caused by the matrix and fiber components of the boom.

#### 3.2.1. Boom Bending Simulation

Firstly, to capture quasi-static linear elastic bending, the FEM model was developed in Abaqus/Implicit for the four-point bending setup as described in Section 2.3, using linear–elastic material properties (shown in Table 1). The model consisted of 18,560 linear quadrilateral shell elements (S4R), representing ply thickness with 1 mm mesh size. The choice of mesh size was determined through a mesh convergence approach. Since the boom was modeled using shell elements, a two-ply laminate was assigned to the flat region and a single-ply laminate to the remaining region. Surface–surface contact was activated for interactions between the rigid roller and the boom, and self-contact was assigned within the area of the lenticular section. The bottom set of rollers was fixed, while the top set provided displacement to apply 2% strain on the UTHS boom.

**Figure 5:**
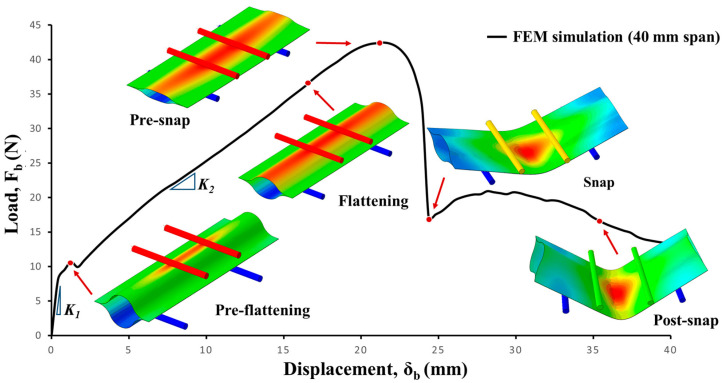
Load–displacement plot of a large deformation structure under pure bending (colors represent vertical displacement).

The modeling of PDA was developed to predict the damage initiation and evolution behavior of the UTHS boom under bending. An explicit modeling approach was chosen to capture the snapping behavior during bending. The calibrated CDM material model was implemented in the 3D (solid) model of the UTHS boom using Abaqus/Explicit. The entire model was defined with a single-ply weave orientation, with thickness being incorporated into the 3D solid model. Cohesive elements were not employed at the flat interface of plies, as no signs of delamination were observed based on experimental testing. Therefore, it was assumed that the inter-adhesion of the ply exhibited linear elastic behavior. The model consisted of 24,780 continuum shell (SC8R) elements, with a mesh size of 1 mm. For a quasi-static bending using dynamic explicit analysis, time, mass scaling parameters, and mesh size were selected to keep the kinetic energy a smaller fraction (5%) of the external work. A displacement rate was assigned to the top pair of rollers to apply 2% strain to the UTHS boom within the given step time. The bottom set of rollers were fixed, similar to the implicit analysis. As shown in Figure 5, major deformation occurred in the region between the top two rollers during high deformation bending. A damage process zone measuring 20 mm in length and 62 mm in width (comprising 2480 elements and 19,842 nodes) was selected to analyze the distribution of damage of the UTHS boom under bending.

#### 3.2.2. Boom Buckling Simulation

The same Abaqus/Explicit model used bending was employed for the simulation of boom eccentric buckling with SC8R elements and 1 mm mesh size. The rationale for choosing this modelling approach was to account for high-order geometric non-linearity, which involves capturing the true experimental scale lenticular cross-section (as depicted in Figure 1a) and boom length (as shown in Figure 3d) without any simplification. We believe by adopting this approach, the explicit solver effectively considers the P-delta effect [29,30] in eccentric buckling. Additionally, the approach addresses structural non-linearity using of a dynamic explicit solver to accurately determine changes in cross-section and structural deformation. Material non-linearity using CDM material model was also incorporated to FEM simulation accuracy. To replicate the end boundary condition of eccentric buckling, a flat rigid shell element was fixed to the boom ends, simulating a fixed boundary with zero slope. The upper flat shell was assigned rotating degrees of freedom along the X-axis (as depicted in Figure 3d) and a downward displacement of 8 mm along the Z-axis. Meanwhile, the lower flat shell had fixed displacement and rotation, excluding rotation along the X-axis. For quasi-static bending, mass scaling was employed, and the step time was adjusted to ensure kinetic energy constituted a smaller fraction (5%) of the external work.

## 4. Analysis of Pure Bending Deformation Modes and Eccentric Buckling Collapse in Composite Boom

The linear–elastic model used in implicit simulation was able to accurately capture all deformation features observed during the test (shown in Figure 5). The load–displacement plot of the implicit analysis shows various deformation modes of the UTHS boom during pure bending. The bending of the UTHS boom began with pre-flattening stage, where the structure undergoes bending with indentation. The phenomenon of bending with indentation was also observed in the three-point bending of a thin-walled rectangular beam [31], as the ratio of top roller distance to beam height ranged between 3 and 7. Compared to the flattening stage, the pre-flattening stage exhibited a stiffer behavior (as shown by the slope (*K*_1_ = 2.96 N/mm, *K*_2_ = 1.64 N/mm) in the load–displacement plot depicted in Figure 5). During flattening, the lenticular cross-section of the boom was narrowed down until it reached a point of pre-snap, at which the load–displacement curve exhibited a plateau. The boom behaved linearly, with an increase in load until the pre-snap phase, at which point the boom achieved its maximum load. At the snap, a rapid decrease in load was observed, and the sample transformed into a folded shape, where the top and bottom surfaces of the boom between the top rollers were pressed together. As the applied displacement increased in the post-snap phase, the folded boom region expanded outwards from the top rollers into the span direction. During this mode of deformation, the load displacement showed plateau-like behavior with the initial stiffening (up until 28 mm) followed by minor softening.

**Figure 6:**
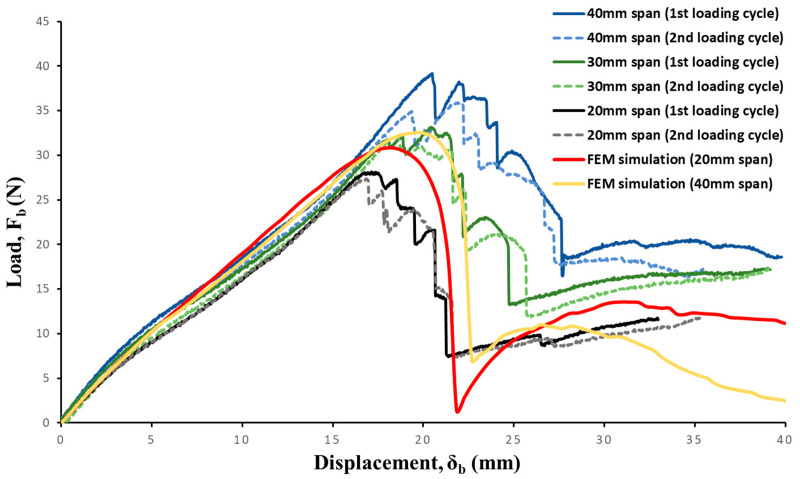
Load–displacement plots for experimental and FEM simulated results for varying effective span lengths.

Figure 6 also presents a comparison between the experimental and implicit simulation results in a load vs. deformation plot. During the experimentation, an 11 N preload was applied to ensure proper alignment of the sample and prevent the boom from rolling before the displacement of the top roller commenced. Consequently, the initial slope (*K*_1_) in the load–displacement curve observed in the FEM simulation, attributed to bending with indentation, was not accounted for in the experimental load–displacement plot. To rectify this, the preload was subtracted from the load–displacement curve obtained through the FEM simulation.

The slope of the flattening stage of the simulation (*K*_2_ = 1.76 N/mm) was observed to be consistent with the experimental results (*K*_2_ = 1.64 N/mm); however, FEA overestimated the pre-snap load for the 20 mm span sample and underestimated for the 40 mm span sample. This overestimation/underestimation of pre-snap load can be attributed to the imperfections of the geometry, as snapping is a stability phenomenon that was not accurately capturing it in an implicit FEM simulation. When comparing the load–displacement behavior of FEM simulations for different *l*_1_ (shown in Figure 6), minimal increases in the peak pre-snap load were observed for an increase in top roller span: 30.84 N for 20 mm span and 32.54 N for 40 mm span. Additionally, an increase in the top roller span resulted in a delay of the snap-stage location. Analyzing the effect of increases in the roller span for experimental results revealed similar trends in the peak pre-snap load but at significantly higher factors: 28 N for 20 mm and 38.41 N for 40 mm. As shown in Figure 6, it is evident from the experimental load–displacement plot that the pre-snap load increases with an increase in roller span, *l*_1_. Moreover, a clear delay in the snapping stage was observed for increases in the span.

The experimental test involved two consecutive loadings for the four-point bending setup, and the load displacement of the second loading cycle is also shown in Figure 6. Upon analyzing the second loading cycle with the first loading cycle, minimal degradation of the UTHS boom was observed: the load–displacement slope of remained unaffected, while peak snapping load only reduced by 4.16 N for *l*_1_ = 40 mm. This reduction in the second loading cycle was caused by high deformation bending in the previous cycle. The post snap plateau showed an overall increase with the top span length *l*_1_. To gain a more comprehensive understanding of the qualitative and quantitative nature of the damage modes, PDA was used.

**Figure 7:**
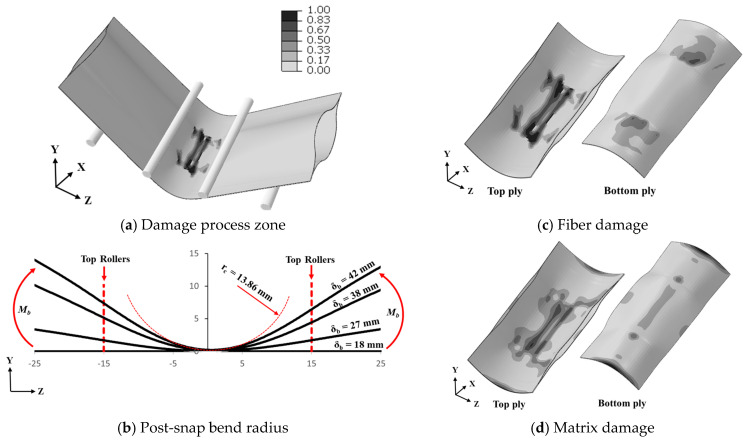
Damage analysis for 30 mm span length.

**Figure 8:**
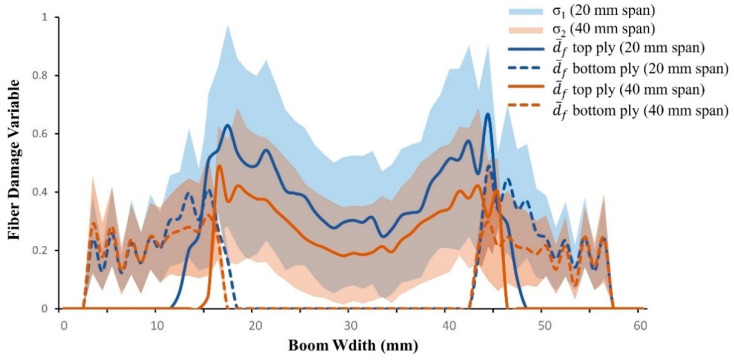
Fiber damage distribution across the boom width in DPZ.

The damage analysis of different span lengths was performed on a UTHS boom using explicit analysis. Quantitative analysis of fiber damage and matrix damage at the damage process zone for the top and bottom ply was performed. The FEM simulation results show that the boom damage process zone undergoes high-strain deformation under snapping, leading to damage initiation and propagation (Figure 7a). The spread of fiber and matrix damage in the damage process zone is presented in Figure 7c,d. The deformation profile of the edge of the lenticular boom is presented in Figure 7b. The deformation profile is shown from pre-snap at roller displacement, *δ_b_* = 18 mm to post-snap *δ_b_* = 42 mm. The post-snap deformed shape was used to calculate the maximum bending radius (*r_c_*) = 13.86 mm at *δ_b_* = 42 mm. The damage was initiated at the snapping-stage and progressed during the post-snap deformation. The concentration of fiber damage distribution (shown in Figure 7c and Figure 8) in the top-ply was observed at the center, while minor damage was found in the bottom ply. The difference in damage distribution between the top and bottom ply was due to the top ply being under compression and the bottom ply being under tensile loads. The top and bottom plies only connected through the contact region; therefore, any crack initiated in the top ply due to compression did not propagate into the bottom ply, as the crack was arrested at the free surface of the top ply. Figure 7c displays a visual distribution of fiber damage, while Figure 8 shows the mean fiber damage, ${\bar{d}}_{f}$, across the boom width and corresponding standard deviation for the top and bottom ply. Similarly, more matrix damage was observed in the top ply and less damage developed in the bottom ply during snapping: Figure 7d shows a visual matrix distribution of the top and bottom ply.

**Figure 9:**
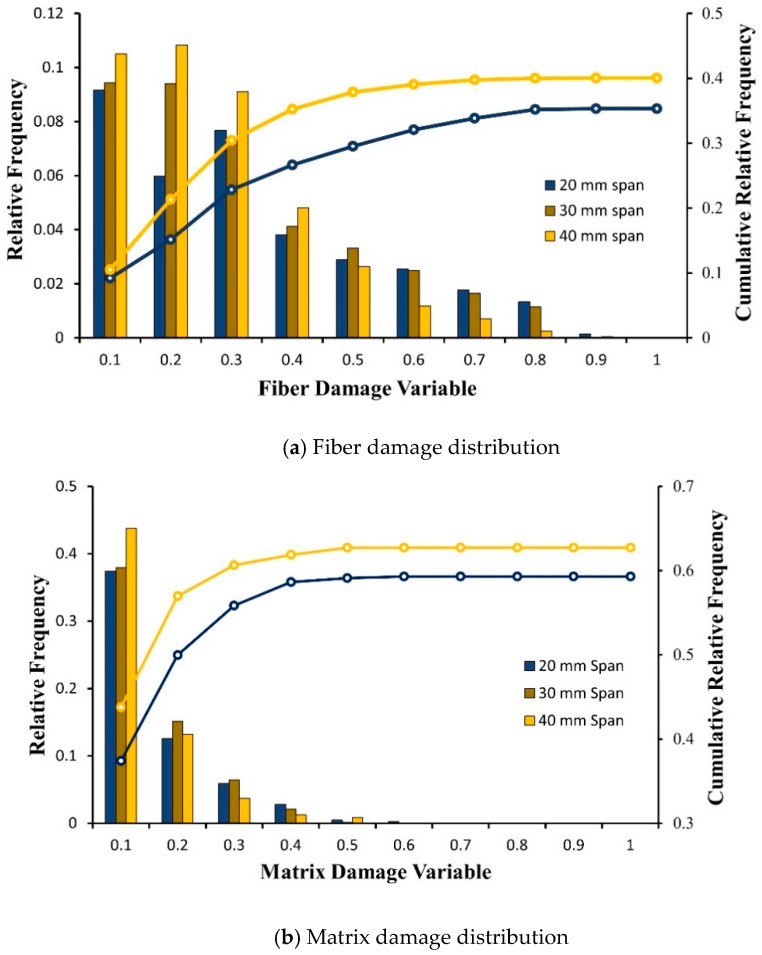
FEA results of fiber and matrix damage comparative analysis for different span lengths.

The analyses of damage for various span lengths were conducted by determining the relative damage frequency and cumulative relative frequency of fiber and matrix damage variables in the damage process zone. The results are presented in Figure 8 and Figure 9, which display the damage variable for different span lengths of the UTHS boom. In Figure 8, it was observed that the mean and standard deviation (damage variation) of fiber damage distributions were higher for the 20 mm span when compared to the 40 mm span. A higher damage was observed because the damages were more concentrated over for a shorter span.

The cumulative relative frequency plots of the matrix and fiber damage in top and bottom plies is shown in Figure 9. Booms with the *l*_1_ = 40 mm span in comparison to *l*_1_ = 20 mm span (Figure 9a) indicated a slightly higher number of elements with fiber damage: 10% vs. 8%. Specifically, there were more damaged elements with low fiber damage variable (between 0.1–0.3). This result explains the marginal reduction in the peak load of the boom after the first loading cycle (Figure 6) for *l*_1_ = 40 mm span, which can be attributed to the fact that for shorter spans, the damage was highly concentrated and less dispersed. Overall results indicate that the damage in the boom was not significantly changed for span lengths ranging from 20 mm to 40 mm for both fiber and matrix.

**Figure 10:**
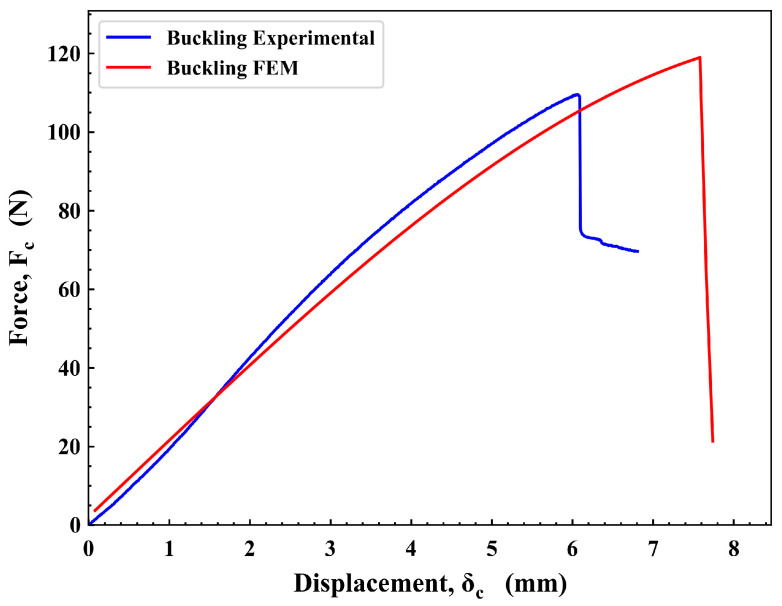
Force–displacement plot from the eccentric buckling test.

The PDA model was further utilized to analyze the damage in the UTHS boom under eccentric buckling load. The eccentric buckling performance was assessed based on force (*F_c_*)–displacement (*δ_c_*) plot. As depicted in Figure 10, the experimental results revealed an initial buckling stiffness of 18.9 N/mm, which momentarily increased and progressively decreased to 11.9 N/mm until failure. The FEM simulation accurately captured this behavior, initiating with a stiffness of 18.8 N/mm and steadily declining to 10.7 N/mm until failure. In terms of strength, the FEM simulation slightly outperformed the experimental results, at 118 N compared to 109 N.

**Figure 11:**
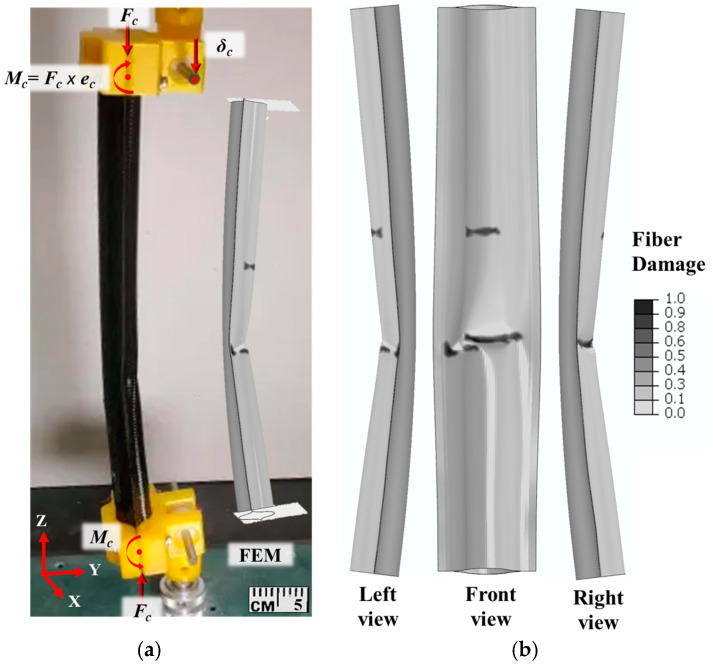
(**a**) Bending collapse comparison: experiment vs. finite element simulation, (**b**) fiber damage distribution in three views under eccentric buckling.

A bending collapse with non-linear behavior (as depicted in Figure 11a) was observed, indicating a transverse crack due to fiber damage. Upon visual inspection, fiber failure was observed in the post-mortem local dimple region; thus, the overall structure underwent global buckling. The PDA model aligned with the visual inspection, revealing fiber failure in the two center regions of the boom, as illustrated in Figure 11b. The major fiber failure occurred on the compressive side of the boom, indicating similar behavior to four-point bending results, which also showed the majority of damage on the compressive side of the flattened boom (Figure 7).

## 5. Conclusions

In conclusion, the fabrication process, structure characterization, and PDM of the UTHS composite boom with a lenticular cross-section was presented for small satellite payloads. The study employs a scalable lenticular boom fabrication process to achieve uniform thickness and presents the challenges encountered during the development of this process. The structure characterization was performed to investigate:

- Large deformation analysis during localized pure bending to determined critical bending radius, crucial for understanding folding deformation and damage during rolling.
- The eccentric buckling test assessed coupled bending and compression behavior, revealing global structural bending under post-deployment loading conditions.

The four-point bending test revealed four stages—flattening, pre-snap, snap, and post-snap—which were effectively captured by the FEA’s modelling approach. The two consecutive bending cycles with different top roller spans revealed sub-critical damage, as validated by the PDA model for a critical bending radius (*r_c_*) of 13.86 mm. The PDA revealed differences in damage distribution between the top and bottom plies due to compression and tension. The FEA model was also able to capture the bending collapse under eccentric buckling load with clear resemblances of the transverse crack fiber damage in the boom. Upon correlating these mechanical tests, the bending capability under pure bending was found to be 0.78 Nm, while under coupled bending and buckling load (*M_c_*), it measured 3.98 Nm. The validated PFA model was able to predict the non-linear global buckling of the boom under eccentric loading, while capturing the complex failure behavior. These mechanical tests facilitated the characterization of the UTHS boom to determine load-bearing capacity for conditions both pre-deployment and post-deployment.

The study’s results have practical implications for the design and manufacturing of ultra-thin composite booms for small satellite applications. The post-snap bend radius during the four-point bending test provides a valuable metric for determining the wrapping radius that can be achieved without significant mechanical degradation, which can be used in achieving a high payload packaging efficiency of composite booms in small satellite systems. The coupled bending-compressive deformation showed the global buckling, which resulted in transverse fiber cracking on the compressive side of the boom. Therefore, the modeling of boom PDM can serve the purpose of structural dynamic analysis for complex deployments and post deployment, aiding in the assessment of fiber and matrix damage. The progressive damage modeling is crucial for designing booms for intricate loading conditions applied in the different new-age deployment of payloads for small satellites and space structures.

## Figures and Tables

**Table 1:** CFRP twill weave fabric mechanical properties.

Symbol	Material Constants (Units)	Magnitude
	Elastic properties	
*E*_1_	Warp Young’s modulus (GPa)	36.90
*E*_2_	Weft traction Young’s modulus (GPa)	32.60
*V*_12_	Poisson coefficient	0.053
*G*_12_	Shear modulus (GPa)	1.560
Strength properties (damage initiation coefficients)		
*X*_1_	Warp strength (MPa)	240
*X*_2_	Weft strength (MPa)	234
*S*	In-plane shear damage threshold (MPa)	15
Fracture toughness (damage evolution coefficients)		
*Gf* ^1+^ *_f_*	Energy rate per unit area warp tension (mJ/mm^2^)	20
*Gf* ^1−^ *_f_*	Energy rate per unit area warp compression (mJ/mm^2^)	40
*Gf* ^2+^ *_f_*	Energy rate per unit area weft tension (mJ/mm^2^)	10
*Gf* ^2−^ *_f_*	Energy rate per unit area weft compression (mJ/mm^2^)	20
Shear damage and hardening parameters (shear plasticity coefficients)		
*α*_12_	Parameter for in-plane shear damage	0.316
*α^max^*_12_	Maximum in-plane shear damage	1
$\sigma_{y}^{0}$	Initial effective shear yield stress (MPa)	25
*C*	Coefficient in the hardening equation	500
*P*	Power term in the hardening equation	0.42

## Data Availability

Data are contained within the article.
